# Hypoxia preconditioning promotes bone marrow mesenchymal stem cells survival by inducing HIF-1α in injured neuronal cells derived exosomes culture system

**DOI:** 10.1038/s41419-019-1410-y

**Published:** 2019-02-12

**Authors:** Zucheng Luo, Fangfang Wu, Enxing Xue, Linlin Huang, Ping Yan, Xiaoyun Pan, Yulong Zhou

**Affiliations:** 1 Department of Orthopaedics, The Second Affiliated Hospital and Yuying Children’s Hospital of Wenzhou Medical University, Wenzhou, Zhejiang 325000 P. R. China; 2 Zhejiang Provincial Key Laboratory of Orthpaedics, Wenzhou, Zhejiang 325000 P. R. China; 3 The Second School of Medicine, WenZhou Medical University, Wenzhou, Zhejiang 325000 P. R. China; 4 Emergency Department, The Second Affiliated Hospital, Wenzhou Medical University, Wenzhou, Zhejiang 325000 China

## Abstract

Bone marrow derived stem cells (BMSCs) transplantation are viewed as a promising therapeutic candidate for spinal cord injury (SCI). However, the inflammatory microenvironment in the spinal cord following SCI limits the survival and efficacy of transplanted BMSCs. In this study, we investigate whether injured neuronal cells derived exosomes would influence the survival of transplanted BMSCs after SCI. In order to mimic the microenvironment in SCI that the neuronal cells or transplanted BMSCs suffer in vivo, PC12 cells conditioned medium and PC12 cell’s exosomes collected from H_2_O_2_-treated PC12 cell’s culture medium were cultured with BMSCs under oxidative stress in vitro. PC12 cells conditioned medium and PC12 cell’s exosomes significantly accelerated the apoptosis of BMSCs induced by H_2_O_2_. Moreover, the cleaved caspase-3, cytochrome (Cyt) *C*, lactate dehydrogenase (LDH) releases, and apoptotic percentage were increased, and the ratio of Bcl-2/Bax and cell viability were decreased. Inhibition of exosome secretion via Rab27a small interfering RNA prevented BMSCs apoptosis in vitro. In addition, hypoxia-preconditioned promoted the survival of BMSCs under oxidative stress both in vivo after SCI and in vitro. Our results also indicate that HIF-1α plays a central role in the survival of BMSCs in hypoxia pretreatment under oxidative stress conditions. siRNA-HIF-1α increased apoptosis of BMSCs; in contrast, HIF-1α inducer FG-4592 attenuated apoptosis of BMSCs. Taken together, we found that the injured PC12 cells derived exosomes accelerate BMSCs apoptosis after SCI and in vitro, hypoxia pretreatment or activating expression of HIF-1α to be important in the survival of BMSCs after transplantation, which provides a foundation for application of BMSCs in therapeutic potential for SCI.

## Introduction

Spinal cord injury (SCI) is still one of the most devastating events with several disabilities and sequelae for individuals^1–3^. Fortunately, with the development of cellular treatments, stem cell-based therapy for SCI has been put proposed as a promising strategy^4,5^. Bone marrow mesenchymal stem cells (BMSCs), because of their unique properties for comparatively easy obtain, rapid proliferative capacity, multipotency, has proved to be a promising candidate for transplantation therapy to SCI^6,7^. Nevertheless, there are a series of obstacles to overcome, particularly the poor cell survival of transplanted stem cells limits their therapeutic efficacy. Thus, finding alternative approaches and exploration of the mechanisms underlying BMSCs in SCI is urgently required. Recent data are mentioned that hypoxia preconditioning, genetic modification, and improving host tissue environment can improve the survival of engrafted cells^8–10^. Indeed, the stem cell-based therapies for SCI have been widely studied. BMSC also secretes extracellular vesicles as a cell-free therapy for nerve injury^11,12^. Furthermore, previously study has demonstrated that culture glial or neuron cells were found to secrete exosomes^13–16^. However, the mechanisms of the SCI-derived exosomes affecting the survival of transplanted BMSCs after SCI remain unknown.

Exosomes represents a subfamily of extracellular vesicles (EVs) with an approximate diameter of 40–200 nm, which as a vital paracrine mechanism in cell–cell communication have been highly focused on^17–19^. Although the function of exosomes had not yet been known, further researches have suggested that they are effective in multiple cellular pathways, as well as pathogenesis of extensive diseases including cardiovascular diseases^20^, neurodegenerative diseases^21^, and human malignancies^22^. In the nervous system, previously studies indicate that exosomes could serve a protective role. For example, they guide axonal development and modulate synaptic activity, which secreted of proteins and RNAs may be a basic mode of communication within the nervous system^23,24^. In contrast, exosomes can also be harmful, they are capable of incorporating of caspase-1 into EVs produced by monocytes and with transfer to surrounding cells^25^. Recent evidence has indicated that Rab27a, which is one of Rab isoforms, are associated with secretory vesicles and involved in the regulation of exocytosis^26^. Rab27a is endogenously expressed in certain neuroendocrine cells^27^, silencing Rab27a in PC12 cells significantly decreased the number of dense-core vesicles docked to the plasma membrane without altering the kinetics of individual exocytotic events^28^. Hypoxic preconditioning (HP) in stem cells is a protective mechanism that has been studied for their ability to promote the efficacy of transplanted cells in certain diseases such as cerebral infarction^29^ and SCI^8^. HIF-1α is one of hypoxia-inducible factor (HIF) of nuclear transcription factor, and plays an important role in hypoxia. The regulatory and active subunit of HIF, often affected by oxygen concentration^30^.

In the present study, we have aimed to investigate whether SCI-derived exosomes could affect the survival of transplanted BMSCs in vivo and vitro. In addition, we investigated if the hypoxic preconditioning improves the survival of BSMCs and the involvement of HIF-1α in survival of BMSCs in hypoxic conditions by using HIF-1α inhibitor and inducer respectively.

## Results

### Oxidative stress caused apoptosis in PC12 cells and BMSCs

**Fig. 1:**
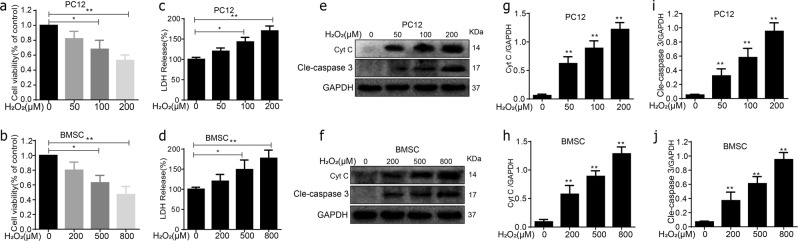
Oxidative stress caused apoptosis in PC12 cells and BMSCs. **a**, **b** H_2_O_2_ induced apoptosis in PC12 cells and BMSCs 24 h posttreatment. **c**, **d** PC12 cells and BMSCs exhibited release of LDH in a dose-dependent of H_2_O_2_ stimulating concentration. **e**–**j** The protein expressions of cleaved caspase-3 and cyt C were visualized by western blot. All data in the figures represent the mean ± SD. *P < 0.05; **P < 0.01, compared with each group, *n* = 5 for each group

The PC12 cells and BMSCs were exposed to different concentrations of H_2_O_2_ for 24 h to mimic the oxidative stress microenvironment after SCI in vivo, followed by the MTT analysis, and cell viability was calculated as percentage of the control group (Fig. 1a, b). Additionally, PC12 cells and BMSCs exhibited release of LDH (lactic acid dehydrogenase) in a dose-dependent of H_2_O_2_ stimulating concentration. Cells were subsequently harvested for protein collection and subjected to western blot analysis. PC12 cells and BMSCs apoptosis were positively related to H_2_O_2_ concentration, as showed by the elevated cleaved caspase-3 and cyt C expression (Fig. 1e–j). Two hundred 200 micrometre H_2_O_2_ caused severe damage to PC12 cells, therefore 100 μM was selected as the stimulating concentration. In addition, compared with the control, when using 200, 500, and 800 μM H_2_O_2_ treatments of BMSCs, a decrease in BMSCs survival was observed (*P* < 0.05; Fig. 1b). As injury of 800 μM H_2_O_2_ displayed too aggressive in promoting cell damage, so choose the concentration of 500 μM to stimulate BMSCs.

### Apoptosis of BMSCs was accelerated in PC12 cells conditioned medium under oxidative stress

**Fig. 2:**
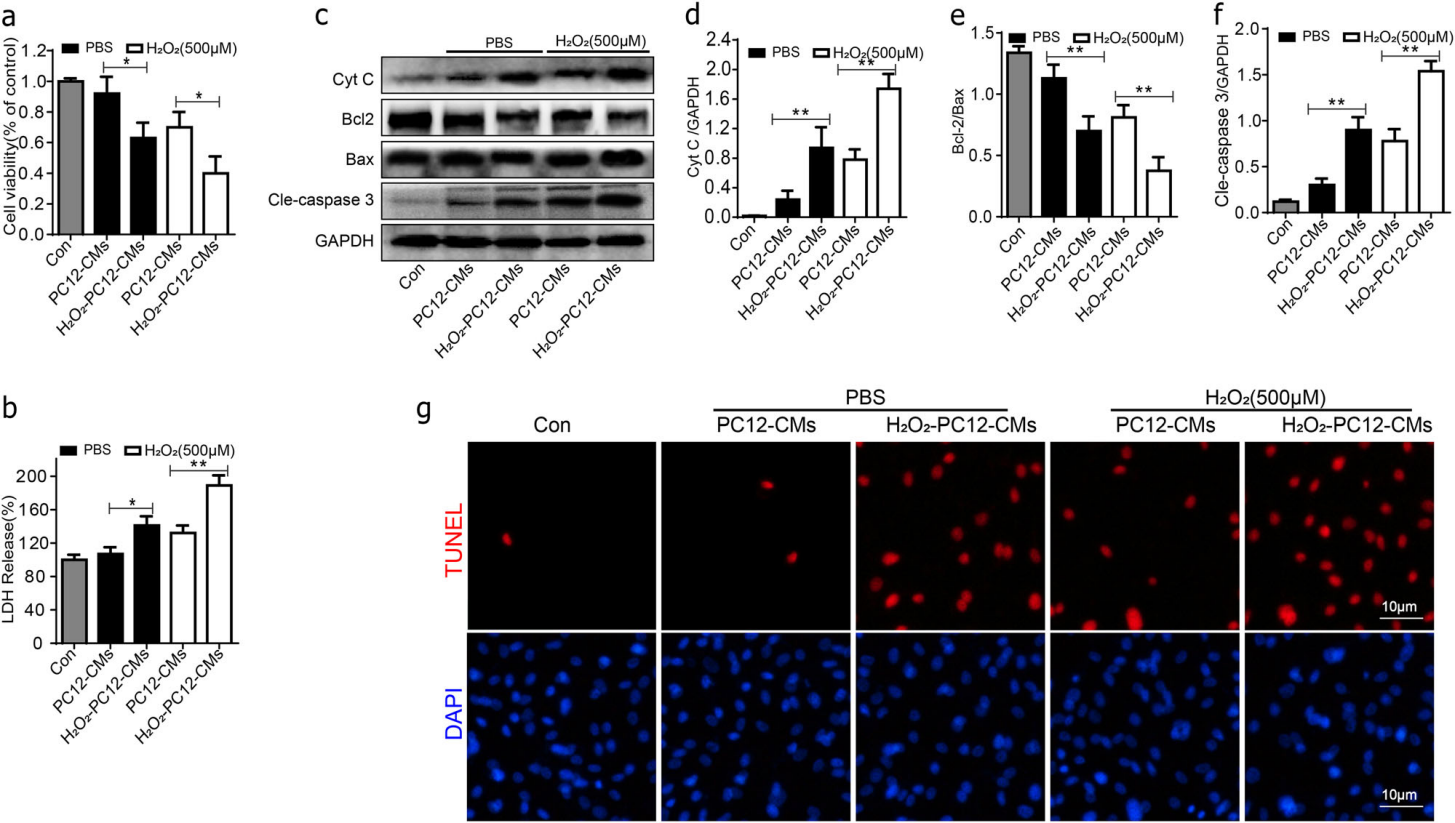
Apoptosis of BMSCs in PC12 cells conditioned medium under oxidative stress. BMSCs were cultured with PC12 cells conditioned medium and treated without or with 500 μM H_2_O_2_ for 24 h. **a** Cell viability of BMSCs in each group was detected by MTT assay. **b** The ratio of LDH release in the group of H_2_O_2_-PC12-CMs exhibited a significantly increased compared to PC12-CMs group treated without or with 500 μM H_2_O_2_ for 24 h. **c**–**f** Representative western blots and quantification data of Cyt C, Bcl-2, Bax, cleaved caspase-3 in each group cells. **g** Double staining for TUNEL (red) and DAPI (blue) of BMSCs in each group cells. All data in the figures represent the mean values ± SD. *P < 0.05; **P < 0.01, compared with each group, *n* = 5

To explore the mechanism of poor survival of BMSC cells implanted in the context of SCI, and whether injured central nerve cells may affect the survival of implanted BMSCs through paracrine action. We cultured BMSCs with PC12 cells conditioned medium under 500 μM H_2_O_2_ for 24 h. Cell viability was greatly decreased in H_2_O_2_-PC12-CMS group compared with PC12-CMs group both in PBS or H_2_O_2_ conditions (Fig. 2a). It significantly changed in cleaved caspase-3 and Cyt c expression between group PC12-CMs and H_2_O_2_-PC12-CMs both in PBS or H_2_O_2_ (Fig. 2c–f). Western blot revealed that H_2_O_2_-PC12-CMs group exhibited a significantly decreased ratio of Bcl-2/Bax compared to PC12-CMs group both in PBS or H_2_O_2_ conditions (Fig. 2c–e). In addition, groups of H_2_O_2_-PC12-CMs exhibited a significantly increased ratio of LDH release compared to PC12-CMs group (Fig. 2b). Moreover, the percentages of TUNEL^+^ cells were significantly higher in H_2_O_2_-PC12-CMs compared to PC12-CMs both in PBS and H_2_O_2_ conditions for 24 h.

### Identification of the PC12 cell’s exosomes and BMSCs

**Fig. 3:**
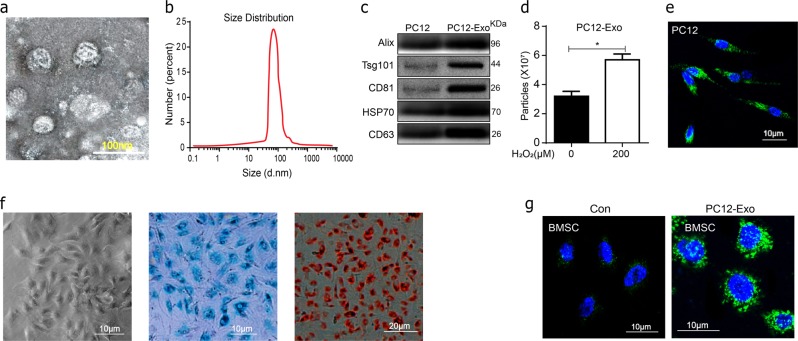
Characterization of PC12 cells derived exosomes. **a** Representative transmission electron microscopy (TEM) images of exosomes. Scale bar 100 nm. **b** Size distributions of exosomes were measured using nanoparticle tracking analysis (NTA). **c** Western-blotting analysis of exosomal markers including CD63, HSP70, CD81, Tsg101, and Alix isolated from PC12 cells derived exosomes. **d** Recording of exosome concentrations from conditioned medium from PC12 cells with or without 100 μM H_2_O_2_ for 24 h by NTA. **e** The PKH67-labeled PC12 cells exosomes and stained with DAPI. **f** Passage three of BMSCs were assessed for their differentiation ability based on Alizarin red staining and alcian blue staining respectively. **g** Uptake of PC12 exosomes by BMSCs. BMSCs were cultured with PKH67-labeled PC12 exosomes or control medium for 24 h and stained with DAPI. All data represent mean values ± SD. *P < 0.05; **P < 0.01, compared with each group

Briefly, the identification of the morphology and properties of isolated particles was confirmed by transmission electron microscopy (TEM), The effective diameter of exosomes was found to be 30–100 nm (Fig. 3a), the concentration and size distribution of the particles was defined by nanoparticle tracking analysis (NTA) (Fig. 3b–d). The presence of exosomal marker CD63, HSP70, CD81, Tsg101, and Alix expression in the exosomes was confirmed by western blot (Fig. 3c). A total of 3.45 ± 0.29 × 10^7^particles per ml was found in con group and 5.75 ± 0.34 × 10^7^ in H_2_O_2_ group (Fig. 3d). Furthermore, PKH67-labeled particles were expressed in PC12 (Fig. 3e). Passage three of BMSCs, which were 70–80% confluent, were assessed for their differentiation ability. They were found to have osteogenic, and chondrogenic differentiation ability based on Alizarin red staining and alcian blue staining respectively. These positive results are showed in Fig. 3f. Furthermore, the result of immunofluorescence showed that after cultured for 24 h the PC12 cell’s exosomes (green fluorescence) were colocalized in BMSCs (Fig. 3g). Therefore, the above results suggested that the PC12 cells derived particles collected in our experiments were exosomes, and that oxidative stress promoted the secretion of exosomes from PC12 cells.

### Exosomes from oxidative stress PC12 cells promote H_2_O_2_-induced apoptosis of BMSCs in vitro

**Fig. 4:**
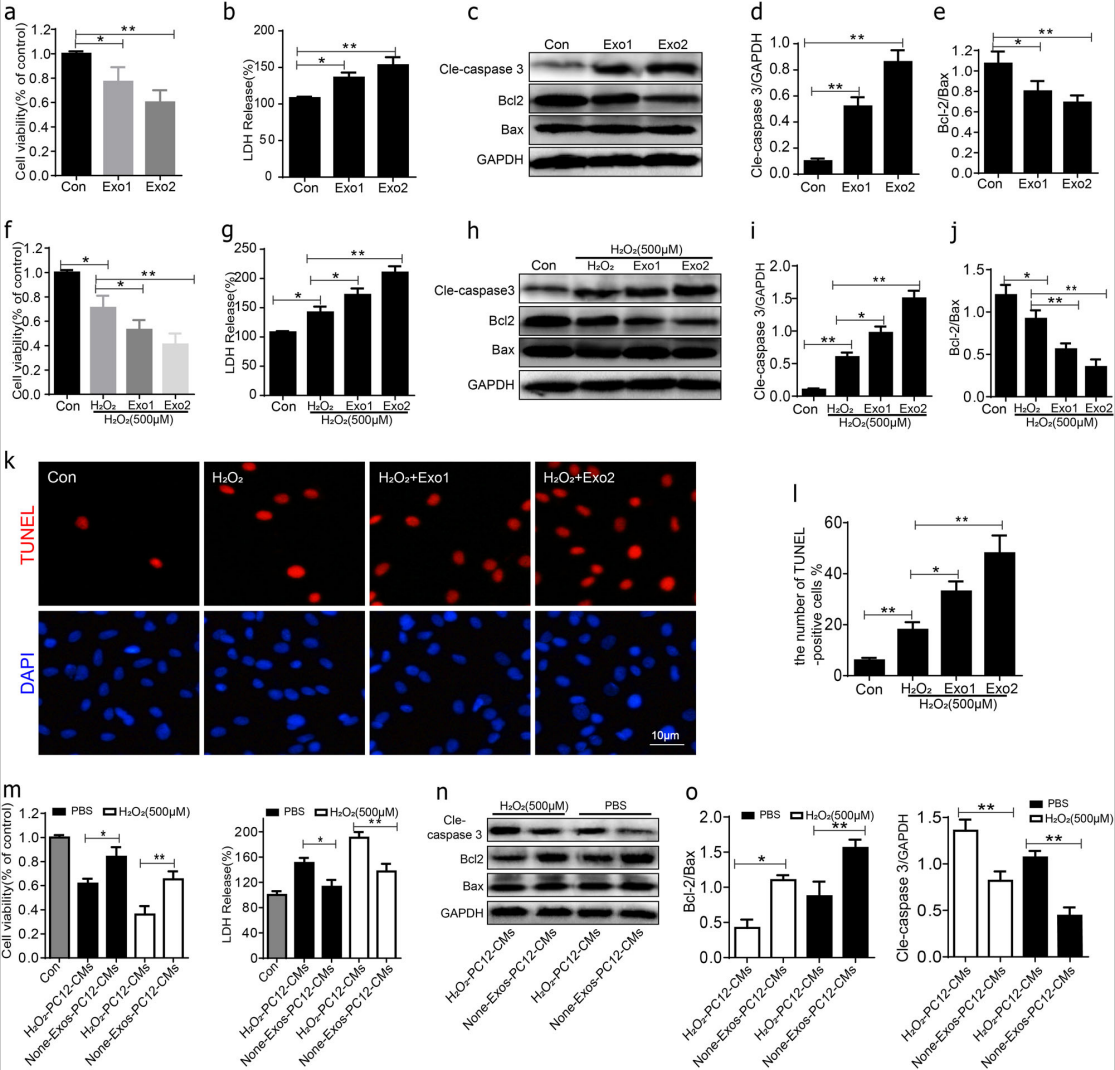
The apoptotic effect of PC12 cells exosomes on BMSCs. **a** Cell viability of BMSCs after being cultured with two different concentrations of PC12 cells exosomes was detected by MTT assay. **b** BMSCs exhibited release of LDH in a dose-dependent of PC12 cells exosomes. **c**–**e** Representative western blots of cleaved caspase-3, Bcl-2, Bax in BMSCs after 24 h culture with PC12 cells exosomes. Quantitative data of cleaved caspase-3/GAPDH and Bcl-2/Bax ratio were shown. **f** Cell viability of BMSCs after being co-cultured with two different concentrations of PC12 cells exosomes and 500 μM H_2_O_2_ for 24 h was detected by MTT assay. **g** BMSCs exhibited release of LDH in a dose-dependent of PC12 cells exosomes under oxidative stress. **h**–**j** Representative western blots and quantification data of cleaved caspase-3, Bcl-2, Bax. **k**, **l** Cell apoptosis assessed by TUNEL staining, the percentages of TUNEL + cells were significantly higher in Exo1 and Exo2 group compared with H_2_O_2_ alone. All data in the figures represent the Mean values ± SD. *P < 0.05; **P < 0.01, *n* = 4, compared with each group

We analyze the regulatory effect of PC12 cell’s exosomes in the BMSCs apoptosis under normal culture or oxidative stress condition. BMSCs were cultured for 24 h with 10% exosomes-depleted fetal bovine serum DMEM containing two different concentrations of exosomes (1, 5 × 10^7^ particles per ml) in normal culture. In MTT and LDH assays, after exposure to exosomes for 24 h, cell viability was reduced in Exo groups, with the increased release of LDH (Fig. 4a, b). Western blot was used to detect the expression of cell apoptosis. As a result of previous results, H_2_O_2_-PC12-CMs promoted H_2_O_2_-induced apoptosis of BMSCs more significantly than other groups. In comparison to the control group, the expression of cleaved caspase-3 and the decrease in Bcl-2/Bax ratio were significantly increased in H_2_O_2_ + Exo groups, depending on the exosomes concentration in normal culture (Fig. 4c–e).

To further investigate the effect of PC12 cell’s exosomes on BMSCs apoptosis under oxidative stress conditions, BMSCs were treated with two different concentrations of PC12 cell’s exosomes conditioned medium and with 500 μM H_2_O_2_ for 24 h. In MTT and LDH assays, after exposure to H_2_O_2_ alone or with exosomes for 24 h, cell viability was significantly reduced in H_2_O_2_ + Exo groups compared with H_2_O_2_ group, accompany with the increased release of LDH (Fig. 4f, g). Then the cell apoptosis was analyzed by western blot and TUNEL staining. The results showed that in the Exos groups, the cleaved caspase-3 was significantly increased and the Bcl-2/Bax ratio was significantly decreased as compared to H_2_O_2_ alone group (Fig. 4h–j). We next performed several times about exosome-free conditioned medium (None-Exos-PC12-CMs) on BMSC death. The results showed that cell viability was significantly increased in None-Exos-PC12-CMs group cells compared to H_2_O_2_-PC12-CMs group (Fig. 4m–o). The percentages of TUNEL + cells were significantly higher in the Exos groups compared to H_2_O_2_ alone group. Moreover, dramatically increased TUNEL + cells were found in the Exo2 group compared with the Exo1 group (Fig. 4k, l). In summary, these results indicated that BMSCs apoptosis could be promoted by oxidative stressed PC12 cells exosomes, which were dependent on the exosomes concentration.

### Inhibition of Rab27a by siRNA reduced the PC12 cell’s exosomes and apoptosis of BMSCs

**Fig. 5:**
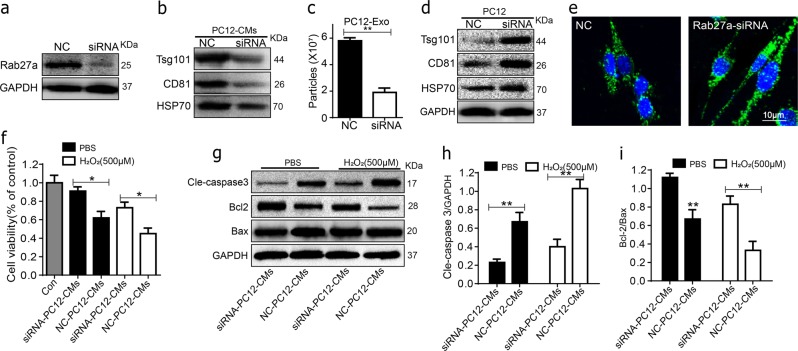
Effect of Rab27a inhibition on the PC12 cells exosomes secretion and apoptosis of BMSCs. **a**, **b** The Rab27a was significantly decreased and presence of exosomal marker HSP70, CD81, and Tsg101 were lower in Rab27a-siRNA group culture mediums as compared with the non-specific control (NC) group assessed by western blot. **c** NTA showed that the number of the secretory exosomes greatly decreased in Rab27a-SiRNA group compared to NC. **d** The expression of exosomal marker HSP70, CD81, and Tsg101 in PC12 cells assessed by western blot. **e** Immunofluorescence of PKH67-labeled the exosomes in PC12 cells. **f** Cell viability of BMSCs in Rab27a-siRNA group and NC-siRNA group mediums both in PBS or H_2_O_2_ conditions were detected by MTT assay. **g**–**i** Representative western blots and quantification data of cleaved caspase-3, Bcl-2, Bax in BMSCs in each group. All data represent Mean values ± SD. *P < 0.05; **P < 0.01, compared with each group

Several studies have shown that Rab27a, which is a member of the Rab family of small GTPases, has a critical role in secretion of exosomes response. To further explore the role of PC12 cell’s exosomes in transplanted BMSCs, the PC12 cells were transfected with Rab27a-siRNA and analyzed by western blot. As shown in Fig. 5a, b, significant decreases of the Rab27a and presence of exosomal marker HSP70, CD81, Tsg101 were lower in the Rab27a-siRNA group culture mediums as compared with the non-specific control (NC) group. Moreover, NTA showed that the number of the secretory exosomes greatly decreased in Rab27a-SiRNA group compared to NC (Fig. 5c). The expression of exosomal marker HSP70, CD81, and Tsg101 was higher in Rab27a-siRNA cells (Fig. 5d). In addition, immunofluorescence was performed to assess the exosomes of PC12 cell. As shown in Fig. 5e mostly exosomes localized in the cytoplasm in Rab27a-SiRNA group compared to NC. To further validate the role of PC12 cell’s exosomes on the transplanted BMSCs survival, BMSCs were incubated with Rab27a-siRNA PC12 cells conditioned medium both in PBS or H_2_O_2_ conditions for 24 h. As shown in Fig. 5f–i, the apoptosis BMSCs and the cleaved caspase-3 was significantly decreased and the Bcl-2/Bax ratio was significantly increased in Rab27a-siRNA group compared to NC-siRNA group both in PBS or H_2_O_2_ conditions. These results suggest that the oxidative stress PC12 cell’s exosomes play an important role in BMSCs apoptosis.

### Hypoxia-preconditioned promotes the survival of BMSCs under oxidative stress

**Fig. 6:**
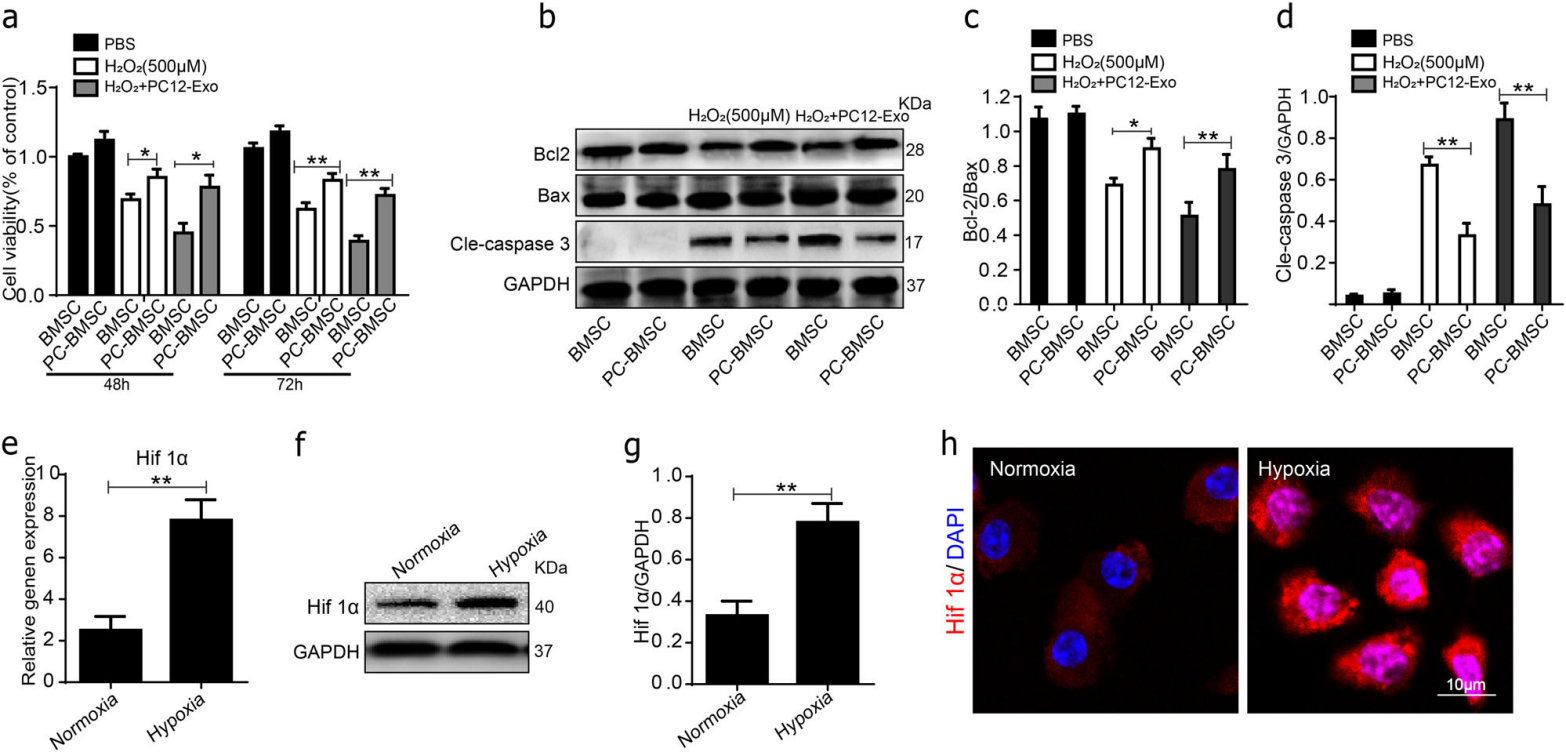
Hypoxia-preconditioned promoted the survival of BMSCs under oxidative stress. **a** Cell viability in the BMSC and PC-BMSC groups cultured in control PBS, H_2_O_2_ (500 μM) and H_2_O_2_ combine with PC12 cell’s exosomes (H_2_O_2_-PC12-Exo) after 48 and 72 h were assessed by MTT. **b**–**d**) Representative western blots and quantification data of cleaved caspase-3, Bcl-2, Bax in BMSCs in each group. **e** The gene expression of HIF-1α in normoxia and hypoxia groups. **f**, **g** Representative Western blots and quantification data of HIF-1α in normoxia and hypoxia groups. **h** Immunofluorescence staining of HIF-1α (red) in normoxia and hypoxia groups, and DAPI - labeled nuclei (blue). *P < 0.05; **P < 0.01, compared with each group, *n* = 5, All data represent Mean values ± SD

The survival of BMSCs could be modified by environmental factors. Among these, hypoxia has been shown to enhance the survival of BMSCs, which may be mediated by hypoxia inducible factor (HIF-1α). To assess whether hypoxia-preconditioned reduced apoptosis of BMSCs. Anoxic preconditioning BMSCs (PC-BMSCs) were cultured with 10% exosomes-depleted FBS DMEM contained PBS, H_2_O_2_ (500 μM) or PC12 cell’s exosomes (H_2_O_2_-PC12-Exo). Cell apoptosis was then assessed by MTT and western blot. As the results shown that the cell viability of BMSCs was improved and cell apoptosis protein cleaved caspase-3 were decreased by hypoxia pretreatment (Fig. 6a–c). As expected, a significant increase in HIF-1α was found in hypoxia group (Fig. 6e–h), which was suggested that hypoxia pretreatment promotes the expression of HIF-1α.

### HIF-1α plays an important role in the survival of BMSCs

**Fig. 7:**
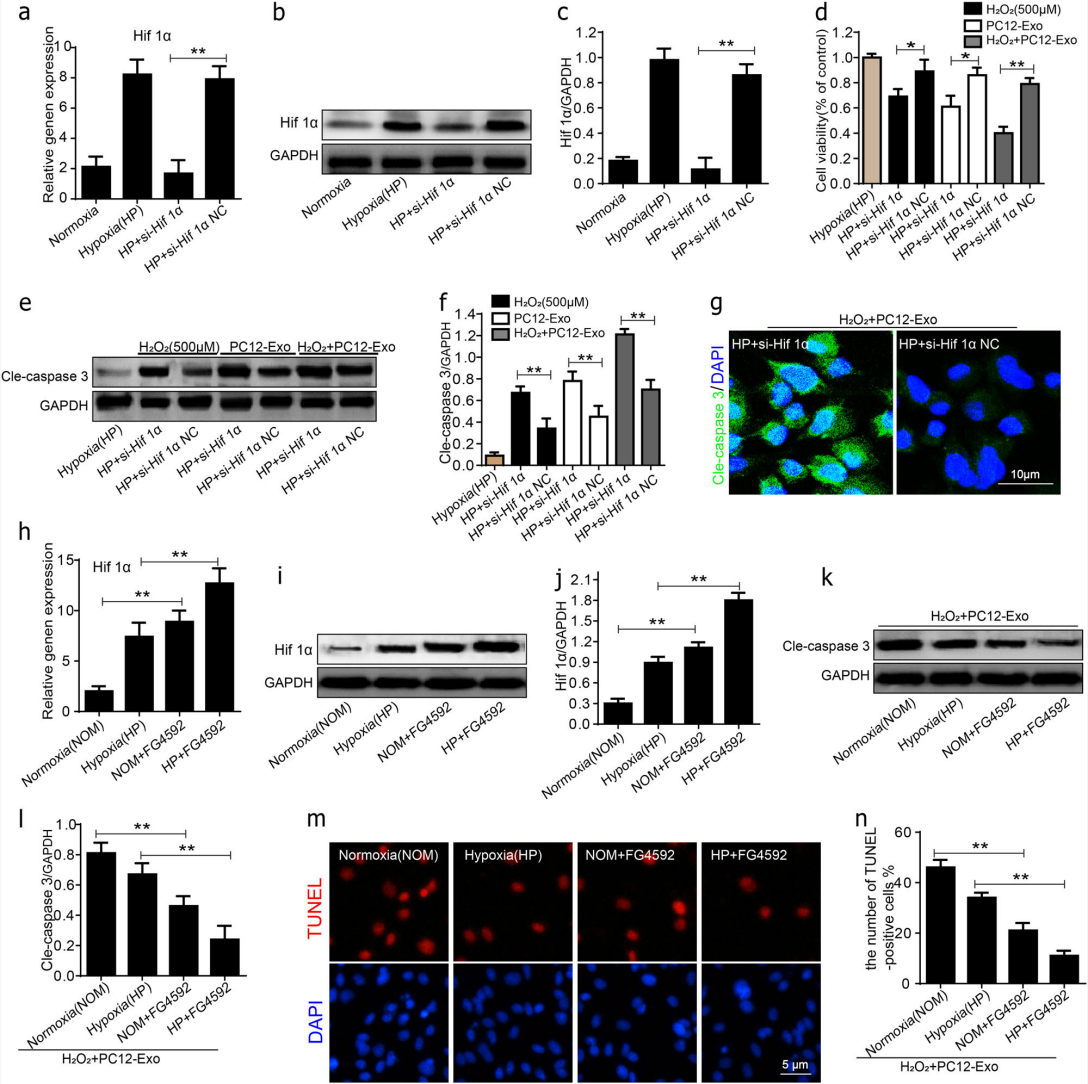
HIF-1α played an important role in the survival of BMSCs. **a**–**c** The gene expression and western blots and quantification data of HIF-1α in the siRNA-HIF-1α group and siRNA-NC groups. **d** BMSCs viability in each group was assessed by MTT. **e**, **f** Representative western blots and quantification data of cleaved caspase-3 in each group. **g** Representative micrographs showing immunofluorescence of cleaved caspase-3 (green) and DAPI - labeled nuclei (blue) in each group. **h**–**j** The gene expression, representative western blots and quantification data of HIF-1α in each group, HIF-1α was up-regulated by using FG-4592. **k**, **l** Representative western blots and quantification data of cleaved caspase-3 in each group. **m**, **n** Cell apoptosis assessed by TUNEL staining in each group. All data in the figures represent the Mean ± SD. *P < 0.05; **P < 0.01, compared with each group

Consequently, to evaluate the potential of HIF-1α in survival-promoting, the BMSCs were transfected with siRNA-HIF-1α and then evaluate the survival of BMSCs in oxidative stress culture condition. The expression of HIF-1α in the siRNA-HIF-1α group was significantly decreased than in the siRNA-NC groups, even after hypoxia (HP) pretreatment (Fig. 7a–c). In MTT assays, cell viability was significantly decreased after HIF-1α-siRNA transfection compared with NC-siRNA group in H_2_O_2_, PC12-Exo, and H_2_O_2_ + PC12-Exo conditions (Fig. 7d). In addition, a significant increase of cleaved caspase-3 was observed in the HIF-1α-siRNA group than the NC-siRNA groups with HP pretreatment in H_2_O_2_, PC12-Exo, and H_2_O_2_ + PC12-Exo conditions (Fig. 7e, f). Furthermore, immunofluorescence staining was performed using anti-cleaved caspase-3 antibodies to evaluate the expression of the cleaved caspase-3 in BMSCs, a significantly increased cleaved caspase-3-positive green fluorescence was observed in the HIF-1α-siRNA group than NC-siRNA group under H_2_O_2_ + PC12-Exo condition. To further study the role of HIF-1α in the survival of BMSCs in hypoxic conditions, HIF-1α was up-regulated by using FG-4592 a cell-permeable prolyl-4-hydroxylase inhibitor in vitro. First, we detected the level of HIF-1α; qPCR and Western blot results showed that the level of HIF-1α expression was much higher after treatment of FG-4592 both in normoxia and hypoxia (Fig. 7h–j). As shown in Fig. 7k, l, the level of caspase-3 was decreased in hypoxic groups, and the decrease were significantly exacerbated in the FG-4592-treated group as compared with the hypoxic groups. We next measured transcripts of HIF target genes of Bcl-2 by RT-PCR, the data showed that the transcription level of the Bcl-2 gene increases with the increase of HIF (Supplementary data. 1). Meanwhile, the result of TUNEL staining was consistent with the Western blot results of caspase-3 (Fig. 7m, n). These results indicate that HIF-1α plays a central role in the survival of BMSCs in hypoxia pretreatment under oxidative stress conditions.

### Hypoxia-preconditioned promotes the survival of BMSCs and improves functional recovery after SCI

**Fig. 8:**
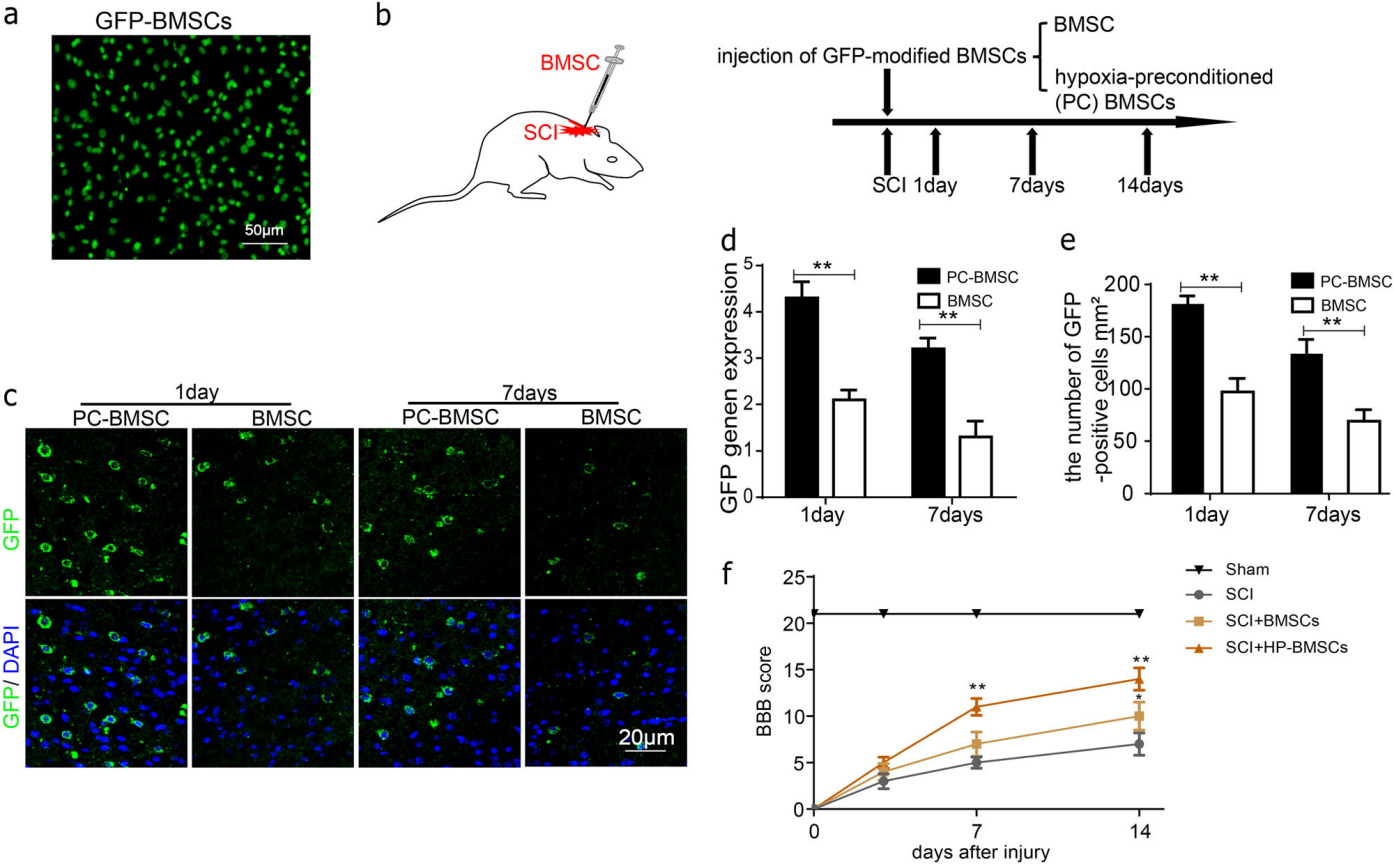
Hypoxia-preconditioned promoted the survival of BMSCs in vivo. **a** All cultured GFP-BMSC emitted sufficient fluorescence for microscopic observations. **b** The GFP-modified BMSCs were transplanted into the spine cord of SCI rats. **c**, **e** Representative micrographs showing immunofluorescence of GFP (green) and DAPI - labeled nuclei (blue) in each group at 1 and 7 day respectively after transplantation. **d** GFP gene expression in the PC-BMSCs groups was significantly increased than in the control groups. All data in the figures represent the averages ± SD. *P < 0.05; **P < 0.01, compared with each group

To further evaluate whether hypoxia-preconditioned can promote the survival of BMSCs in vivo, we transplanted GFP-modified BMSCs into the spine cord of SCI rats (Fig. 8b). Fluorescence microscopy clearly showed that almost all cultured GFP-BMSC emitted sufficient fluorescence for microscopic observations (Fig. 8a). GFP mRNA were used to estimate the survival of implanted cells by RT-PCR. There were more GFP-positive BMSCs survival in the hypoxia-preconditioned BMSCs (PC-BMSC) groups than in the control groups at 1 and 7 days respectively after transplantation (Fig. 8c–e). RT-PCR analysis also showed that the GFP gene expression in PC-BMSCs groups was significantly increased than in the control groups (Fig. 8d). In addition, the therapeutic effect of PC-BMSC was evaluated using Basso, Beattie, and Bresnahan (BBB) score to on SCI. As a result, PC-BMSC treatment significantly increased the locomotor activity 7–14 days after injury, compared with that observed in SCI and BMSC group (Fig. 8f). These data indicated that hypoxia-preconditioned may decrease transplanted BMSCs apoptosis and improves functional recovery after SCI.

## Discussion

**Fig. 9:**
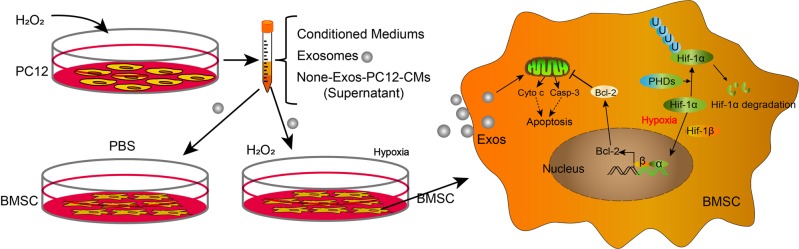
Schematic illustration of the survival of hypoxia-preconditioned BMSCs. Technical and potential molecular mechanism involved in the survival of hypoxia-preconditioned BMSCs

Spinal cord injury (SCI) remains an unresolved problem in medicine^31^. In previous study, stem cells transplantation has become a promising therapy for SCI, while the survival rate of transplanted stem cells is low, limiting the application of spinal cord injury^8,32^. Much of this attrition can be attributed to the hostile microenvironment in the injured area after SCI. The aim of present study was to explore whether the injured neuronal cells have an ability to affect the survival of transplanted cells by paracrine action. We cultured BMSCs with PC12-CMs conditioned medium under oxidative stress in vitro. The result shows that compared to the respective controls the expression of apoptosis-related proteins was significantly increased and cell viability was decreased in the H_2_O_2_-PC12-CMs group both in PBS or H_2_O_2_ conditions. Meanwhile, the group of H_2_O_2_-PC12-CMs in H_2_O_2_ condition was worse than the group of H_2_O_2_-PC12-CMs in PBS condition (Fig. 9). These data suggested that PC12 cells conditioned medium could promote H_2_O_2_-induced BMSCs apoptosis.

Extracellular vesicles (EVs) representing a novel form of intercellular communication has recently emerged in the past few years^33^. Exosomes are the smallest EVs and excreted to the extracellular space by the conjugation of intermediate endocytic bodies to the plasma membrane. Recent advances in knowledge of exosomes including their many roles in cellular communication and human pathologies or treating and diagnosing human diseases. Exosomes produced by stem cells have shown great therapeutic effects in some diseases including ischemia, osteonecrosis and chronic cutaneous wound^34–36^. Recently, several studies have demonstrated that exosomes derived from BMSCs (BMSCs-Exos) could effectively reduce neuronal cells apoptosis, decrease the inflammatory response and promote functional recovery after acute SCI^37^. However, there are short of direct experimental evidence that neuronal cell derived exosomes affecting the transplanted BMSCs after SCI and the underlying mechanism remains enigmatic. Various cells in the nervous system have been shown to release exosomes, suggesting their active effect on the adaptive response of the organisms to stress and maintain homeostasis^23^. We therefore hypothesized whether direct administration of exosomes derived from neuronal cell may overcome the limitations and challenges associated with transplanted stem cell therapy and promote functional outcome after SCI. We speculated that oxidative stressed PC12 cells exosomes have effect on BMSCs apoptosis under normal culture or oxidative stress. In this study, exosomes were obtained from the conditioned culture medium of PC12 cells under oxidative stress, which was used in the culture of BMSCs. The result has shown that the expression of apoptosis-related proteins and apoptotic rates were significantly increased in group H_2_O_2_-Exos. These results indicate that the exosomes in oxidative stressed PC12 cells conditioned medium may have an important role in promoting H_2_O_2_-induced BMSCs apoptosis.

These findings instigated us to further explore the mechanism underlying PC12 cells exosomes accelerated transplanted BMSCs injury. Understanding the transport of exosomes will provide insight into how cells employ these extracellular organelles for intercellular communication^23^. In previously studies, release of exosomes appeared to be dependent on Rab27 and can be blocked by an inhibitor of neutral sphingomyelinase^26^. Silencing Rab27a in PC12 cells significantly decreased the number of dense-core vesicles docked to the plasma membrane of individual exocytotic events^28^. Therefore, we decided to inhibit the Rab27a in PC12 cells and BMSCs apoptosis in the Rab27a-SiRNA group were greatly decreased. As shown in Fig. 5, significantly decreases of the Rab27a and presence of exosomal marker HSP70, CD81, Tsg101 were higher in the Rab27a-siRNA group culture mediums as compared with the non-specific control (NC) group (*P < 0.05, **P *<* 0.01). From the results of our study, inhibiting the expression of Rab27a could decrease exosome secretion and may prevent BMSCs apoptosis in vitro. However, precise signal mechanisms remain to be determined.

Several previous studies have revealed hypothesis that hypoxic preconditioning (HP) prior to transplantation increases the survival rate of BMSCs^38,39^. The potential mechanisms were that HP may promote the secretion of HIF‑1α by BMSCs^40,41^, HIF‑1α is a nuclear transcription factor, the expression of which is upregulated under hypoxic conditions, which can regulate the crucial cellular processes such as stemness, proliferation, and differentiation. However, whether HP could reduce the apoptosis of BMSCs under oxidative stressed neuronal cells derived exosomes conditioned medium deserved further considered. In the present study, we demonstrated HIF-1α overexpression reduced apoptosis of BMSCs in vitro under oxidative stress. In the siRNA-HIF-1α group were observed that the expression levels of HIF‑1α and BMSCs viability were significantly decreased, compared with those of the NC-siRNA group, even after HP pretreatment, in the HIF-1α-siRNA group with HP pretreatment in H_2_O_2_ + PC12-Exo conditions were more obviously, but apoptosis of BMSCs were attenuated in HIF-1α inducer FG-4592 group. Indicating that HP or activating expression of HIF-1α increase the tolerance of BMSCs to harsh microenvironments after transplantation.

In conclusion, our research demonstrated that neuronal cells exosomes accelerate transplanted BMSCs injury after SCI and reveal a potential mechanism for the poor survival of transplanted stem cells. In addition, our results showed that hypoxic preconditioning increases BMSCs survival rate of transplanted BMSCs, and for promoting the protective effects of BMSCs on SCI. Furthermore, HIF-1α play an important role in survival of BMSCs in oxidative stress in vitro. Finally, although further research is still needed, data obtained in this study provide an effective method of improving SCI treatment.

## Materials and methods

### Animals

Ten-week-old male Sprague–Dawley (SD) rats were purchased from Animal Center of Chinese Academy of Sciences Shanghai, China. The protocol for animal care and use was according to the Guide for the Care and Use of Laboratory Animals of the National Institutes of Health and was approved by the Animal Care and Use Committee of Wenzhou Medical University. During the study, the rats had access to bulk food and drinking water adlibitum. The environmental conditions were in accordance with the relevant standards of the Chinese national standard “Environment Control for Experimental Animal Facilities” (GB14925-2001) on barrier animal facilities.

### Experimental SCI model and BMSCs transplantation

The rat model of SCI was induced using an improved impactor based on Allen’s method as previously described^42,43^. Briefly, all animals were anesthetized and injected intraperitoneally with sodium pentobarbital (50 mg/kg), and operated on under aseptic conditions. The incision area was shaved and prepared with betadine solution, chlorhexidine, and sterile water, an incision was made at the thoracic region (T9–T12), and the skin was separated. A laminectomy was performed to expose spinal cord level T10, exposing the dorsal cord surface with the dura remaining intact. The impactor (weighing 10 g, 3 mm in diameter, and 200 mm in length) was dropped from a height of 50 mm to the surface of the spinal cord. Successfully induced SCI resulted in spinal cord congestion, swaying legs, tail swing reflexes, and slow paralysis. The wound was sutured, all animals were kept in a separate environment at 24 °C to ensure adequate water, food, and clean bedding. The rats were provided intermittently with assisted urination 3 times daily.

The adenoviral vector encoding the GFP reporter gene was transduced into the passage 3 male rat BMSCs. Transduction efficiency was assessed by detection of GFP fluorescence signal intensity. GFP-modified BMSCs were detached with 0.125% (w/v) trypsin, suspended in phosphate buffer saline (PBS), and kept on ice until injection. In the injury PC-BMSC group, the animals underwent a laminectomy plus traumatic SCI operation and then were injected with GFP-modified hypoxia-preconditioned (PC) BMSCs of 2.0 × 10^6^ cells (total of 50 μl in PBS) immediately. In the injury BMSC groups, the animals were subjected to similar treatment consisting of an equivalent volume of GFP-modified BMSC.

### Locomotion recovery assessment

The BBB scores were assessed in an open field scale by two blinded independent examiners at 14 days post-operation. Briefly, the BBB locomotion rating scale scores range from 0 points (complete paralysis) to 21 points (normal locomotion). Animals were placed individually in an open field and allowed to move freely for 5 min.

### Cell isolation and culture

BMSCs were isolated from SD rats according to previously established methods^44^. The BMSCs were cultured with DMEM (Gibco, Invitrogen, Grand Island, NY) containing 10% fetal bovine serum (FBS) (Hyclone, Thermo Scientifc, Logan, UT, USA) and 1% antibiotics (Gibco, Invitrogen, Grand Island, NY). Culture medium was replaced every 3 days and the removal of nonadherent hematopoietic cells was performed. To identify the multiple differentiation potential, chondrogenic, osteogenic differentiation kit (Cyagen, Guangzhou, China) was used to induce osteogenic differentiation. The cells were maintained in the chondrogenic, osteogenic induction medium for 14 days and then subjected to Alcian blue and Alizarin red staining. Cells at passage 3 were used for further experiments. Hypoxia preconditioning was performed by an anaerobic chamber (Thermo 1029, California, USA) for 48 or 72 h. The oxygen concentration in the chamber was maintained at 1%, with a residual gas mixture composed of 5% CO_2_ and balanced N_2_. Normal-BMSC were incubated under 21% oxygen and 5% carbon dioxide.

### Western blot

Western blot analysis was performed as previously described^45^. Briefly, proteins were extracted, normalized, and separated by sodium dodecylsulfate-polyacrylamide gel electrophoresis (SDS-PAGE) and transferred to polyvinylidene difluoride (PVDF) membranes. Nonfat milk of 5% in Tris-buffered Saline with Tween 20 (TBST) for 2 h at room temperature was used to block the membranes. The membranes were incubated with primary antibodies overnight at 4 °C, followed by subsequently incubation with respective secondary antibodies for 2 h at room temperature. After 3 times washing with TBST, the detection was performed by electrochemiluminescence plus reagent. Finally, the intensity of these blots was quantified with Image Lab 3.0 software (Bio-Rad).

### Oxidative stress injury in vitro model

To simulate the oxidative stress microenvironment after SCI that the transplanted BMSCs encounter in vivo, the cells were exposed to different concentrations of hydrogen peroxide (H_2_O_2_) in serum-free DMEM for 24 h. The cell viability was detected by use of 3-(4,5-dimethylthiazol-2-yl)-2,5-diphenyltetrazolium bromide (MTT, Sigma, USA) assay according to the manufacturer’s instructions. For the detection of the cleaved caspase-3, cytc, Bax, Bcl-2, protein lysates were prepared and detected by western blot analysis.

### Experiments with conditioned medium in vitro

Conditioned medium (CMs) was prepared as follows: 80–90% confluent PC12 were cultured with serum-free DMEM (PC12-CMs) or serum-free DMEM and subjected to 100 μM H_2_O_2_ treatment (H_2_O_2_-PC12-CMs) for 24 h. Con (without CMs) and PC12-CMs respectively served as control of PC12-CMs and H_2_O_2_-PC12-CMs. The collected culture supernatant was centrifuged at 1500 rpm for 10 min, and was used immediately or after being stored at −20 °C. After the 80–90% confluence, BMSCs were cultured with controlled medium (Con and PC12-CMs) or conditioned medium (PC12-CMs and H_2_O_2_-PC12-CMs) and then mixed with or without 500 μM PBS or H_2_O_2_ for 24 h. Cell viability analysis was detected using MTT. Cell apoptosis was determined by western blot.

### Isolation and characterization of exosomes

The PC12 cells exosomes isolation procedures were performed by differential ultracentrifugation following state-of-the-art protocols^46^. Briefly, 50 ml serum-free DMEM was used for culturing PC12 cells in two T175 flasks. After treatment with 100 μM H_2_O_2_ for 24 h, supernatant was differentially centrifuged at 300 × *g* for 10 min, 2000 × *g* for 10 min, 10,000 × *g* for 30 min, and 100,000 × *g* for 70 min. The final supernatant is then ultracentrifuged at 100,000 × *g* to pellet the small vesicles that correspond to exosomes. The pellet is washed with a large volume of PBS, to remove any residual cells and debris, and centrifuged one last time at the same high speed. Then suspended in 50 μl of sterile PBS and directly used or stored at −80 °C. The concentration and size distribution of exosomes were confirmed by NTA using NanoSight NS300 (Malvern, UK). The differences in exosome concentrations between conditioned medium from PC12 cells mixed with or without 100 μM H_2_O_2_ for 24 h were recorded. The morphology was observed by Transmission Electron Microscopy (TEM, Hitachi H7650 TEM, Japan).

### BMSCs cultured with oxidative stressed PC12 cell’s exosomes

BMSCs were cultured with two different concentrations of exosomes (1, 5 × 10^7^ particles per ml) for 24 h in normal culture (DMEM containing 10% exosomesdepleted FBS). In exploring the effects of PC12 cell’s exosomes on BMSCs apoptosis under oxidative stress, BMSCs were pre-incubated with two different concentrations of exosomes (as previous described) and treated with serum-free DMEM and 500 μM H_2_O_2_ for 24 h. Following treatment, western blot, TUNEL were used to analyze the cell apoptosis, while LDH release and MTT intake were used as cell injury parameters.

### siRNA transfection

The specific Rab27a small-interfering RNA (siRNA) was purchased from Invitrogen (Carlsbad, CA, USA). The sequences of Rab27a siRNA were: sense, 5′-TTCAGGGACGCTATGGGTTT-3′; antisense, 5′-TCCTCTTTCACTGCCCTCTG-3′. Rab27a and negative control of siRNA transfection were undertaken using LipofectamineTM RNAiMAX Reagent according to manufacturer instruction.

### Real-time PCR assay the survival of implanted BMSCs

Total RNA was extracted from cells using TRIzol (Invitrogen). The quantitative real-time PCR (qRT-PCR) experiments were performed for quantification of the GFP-cDNA of BMSCs after 1 and 7 days of cell implantation using SYBR-Green reagents (Takara Bio Inc., Shiga, Japan) with specific primers for GFP (sense, 5′-AAGTTCATCTGCACCACCG-3′; antisense, 5′-TCCTTGAAGAAAGGTGCG-3′) and β-actin (sense, 5′-TGGCTCCTAGCACCATGAAG-3′; antisense, 5′-AACGCAGCTCAGTAACAGTCC-3′). The relative PCR products were calculated with the 2^−ΔΔCt^ method.

### TUNEL and immunofluorescence

The TUNEL experiment was performed using a TUNEL cell apoptosis detection kit (Roche Applied Science, Indianapolis, IN, USA) according to the manufacturer’s protocol. Immunofluorescence (IF) staining was performed as previously described^47^. Briefly, cell slides were incubated with primary antibodies. DAPI was applied to show the nucleus. Representative images were captured with an Olympus IX70 (Olympus, Tokyo, Japan).

### Statistical analyses

All data are expressed as the means ± standard deviation (SD). Comparisons among groups were compared by analysis of variance (ANOVA) or *t*-test, as appropriate. A value of *p* < 0.05 was considered significant. SPSS 20.0 was used to analyze the data.

## Supplementary information

supplemental data

Supplemental figure legends

## References

[1] S. R. Andresen F (2016). Finnerup, pain, spasticity and quality of life in individuals with traumatic spinal cord injury in Denmark. Spinal Cord..

[2] C. S. Rivers N (2018). Health conditions: effect on function, health-related quality of life, and life satisfaction after traumatic spinal cord injury. a prospective observational registry cohort study. Arch. Phys. Med. Rehabil..

[3] S. V. Hiremath NS (2017). Longitudinal prediction of quality-of-life scores and locomotion in individuals with traumatic spinal cord injury. Arch. Phys. Med. Rehabil..

[4] Kwon BK, Tetzlaff W, Grauer JN, Beiner J, Vaccaro AR (2004). Pathophysiology and pharmacologic treatment of acute spinal cord injury. Spine J..

[5] Tykocki T, Poniatowski L, Czyz M, Koziara M, Wynne-Jones G (2017). Intraspinal pressure monitoring and extensive duroplasty in the acute phase of traumatic spinal cord injury: a systematic review. World Neurosurg..

[6] K. Kanekiyo T (2018). Effects of intrathecal injection of the conditioned medium from bone marrow stromal cells on spinal cord injury in rats. J. Neurotrauma.

[7] Neirinck V. et al. Adult bone marrow mesenchymal and neural crest stem cells are chemoattractive and accelerate motor recovery in a mouse model of spinal cord injury. *Stem Cell Res. Ther.* **6**, 211 (2015). 10.1186/s13287-015-0202-2 PMC4632651 26530515

[8] Wang W (2018). Hypoxic preconditioned bone mesenchymal stem cells ameliorate spinal cord injury in rats via improved survival and migration. Int. J. Mol. Med..

[9] Song. JL (2017). Lentivirus-mediated microRNA-124 gene-modified bone marrow mesenchymal stem cell transplantation promotes the repair of spinal cord injury in rats. Exp. Mol. Med..

[10] Cantinieaux D (2013). Conditioned medium from bone marrow-derived mesenchymal stem cells improves recovery after spinal cord injury in rats: an original strategy to avoid cell transplantation. PLoS One.

[11] Shiue, S. J. et al. Cheng, mesenchymal stem cell exosomes as a cell-free therapy for nerve injury-induced pain in rats, *Pain*, 10.1097/j.pain.0000000000001395 (2018). 10.1097/j.pain.0000000000001395 30188455

[12] G. Sun G (2018). hucMSC derived exosomes promote functional recovery in spinal cord injury mice via attenuating inflammation. Mater. Sci. Eng. C Mater. Biol. Appl..

[13] D. Fitzner M (2011). Selective transfer of exosomes from oligodendrocytes to microglia by macropinocytosis. J. Cell. Sci..

[14] Wang G (2012). Astrocytes secrete exosomes enriched with proapoptotic ceramide and prostate apoptosis response 4 (PAR-4): potential mechanism of apoptosis induction in Alzheimer disease (AD). J. Biol. Chem..

[15] Potolicchio I (2005). Proteomic analysis of microglia-derived exosomes: metabolic role of the aminopeptidase CD13 in neuropeptide catabolism. J. Immunol..

[16] J. Faure G (2006). Exosomes are released by cultured cortical neurones. Mol. Cell. Neurosci..

[17] Simons M, Raposo G (2009). Exosomes–vesicular carriers for intercellular communication. Curr. Opin. Cell Biol..

[18] Huang L (2015). Exosomes in mesenchymal stem cells, a new therapeutic strategy for cardiovascular diseases?. Int. J. Biol. Sci..

[19] Kourembanas S (2015). Exosomes: vehicles of intercellular signaling, biomarkers, and vectors of cell therapy. Annu. Rev. Physiol..

[20] M. Hu G (2018). The harsh microenvironment in infarcted heart accelerates transplanted bone marrow mesenchymal stem cells injury: the role of injured cardiomyocytes-derived exosomes. Cell Death Dis..

[21] Ridolfi B, Abdel-Haq H (2017). Neurodegenerative disorders treatment: the microRNA role. Curr. Gene Ther..

[22] Sun, L., et al. Regulation of RAB22A by mir-193b inhibits breast cancer growth and metastasis mediated by exosomes, *Int. J. Oncol.*, 10.3892/ijo.2018.4571 (2018). 10.3892/ijo.2018.4571 30272274

[23] Lai CP, Breakefield XO (2012). Role of exosomes/microvesicles in the nervous system and use in emerging therapies. Front. Physiol..

[24] Smalheiser NR (2007). Exosomal transfer of proteins and RNAs at synapses in the nervous system. Biol. Direct.

[25] Sakar Y (2009). Positive regulatory control loop between gut leptin and intestinal GLUT2/GLUT5 transporters links to hepatic metabolic functions in rodents. PLoS One.

[26] Ostrowski M (2010). Rab27a and Rab27b control different steps of the exosome secretion pathway. Nat. Cell Biol..

[27] Cheviet S, Coppola T, Haynes LP, Burgoyne RD, Regazzi R (2004). The Rab-binding protein Noc2 is associated with insulin-containing secretory granules and is essential for pancreatic beta-cell exocytosis. Mol. Endocrinol..

[28] Tsuboi T, Fukuda M (2006). Rab3A and Rab27A cooperatively regulate the docking step of dense-core vesicle exocytosis in PC12 cells. J. Cell Sci..

[29] Theus MH (2008). In vitro hypoxic preconditioning of embryonic stem cells as a strategy of promoting cell survival and functional benefits after transplantation into the ischemic rat brain. Exp. Neurol..

[30] Meneses AM, Wielockx B (2016). PHD2: from hypoxia regulation to disease progression. Hypoxia.

[31] K. Stephan S (2015). Spinal cord injury–incidence, prognosis, and outcome: an analysis of the TraumaRegister DGU. Spine J..

[32] Vaquero J, Zurita M (2009). Bone marrow stromal cells for spinal cord repair: a challenge for contemporary neurobiology. Histol. Histopathol..

[33] Zaborowski MP, Balaj L, Breakefield XO, Lai CP (2015). Extracellular vesicles: composition, biological relevance, and methods of study. Bioscience.

[34] Tao SC (2017). Exosomes derived from miR-140-5p-overexpressing human synovial mesenchymal stem cells enhance cartilage tissue regeneration and prevent osteoarthritis of the knee in a rat model. Theranostics.

[35] Guo SC (2017). Exosomes derived from platelet-rich plasma promote the re-epithelization of chronic cutaneous wounds via activation of YAP in a diabetic rat model. Theranostics.

[36] Hu GW (2015). Exosomes secreted by human-induced pluripotent stem cell-derived mesenchymal stem cells attenuate limb ischemia by promoting angiogenesis in mice. Stem Cell Res. Ther..

[37] Liu, W., et al. Exosomes derived from bone mesenchymal stem cells repair traumatic spinal cord injury by suppressing the activation of A1 neurotoxic reactive astrocytes, *Journal of Neurotrauma*10.1089/neu.2018.5835 (2018). 10.1089/neu.2018.5835 29848167

[38] Liu H (2012). The role of SDF-1-CXCR4/CXCR7 axis in the therapeutic effects of hypoxia-preconditioned mesenchymal stem cells for renal ischemia/reperfusion injury. PLoS One.

[39] Chang CP (2013). Hypoxic preconditioning enhances the therapeutic potential of the secretome from cultured human mesenchymal stem cells in experimental traumatic brain injury. Clin. Sci..

[40] Kanichai M, Ferguson D, Prendergast PJ, Campbell VA (2008). Hypoxia promotes chondrogenesis in rat mesenchymal stem cells: a role for AKT and hypoxia-inducible factor (HIF)-1alpha. J. Cell. Physiol..

[41] Liu H (2010). Hypoxic preconditioning advances CXCR4 and CXCR7 expression by activating HIF-1alpha in MSCs. Biochem. Biophys. Res. Commun..

[42] Kurnellas MP, Nicot A, Shull GE, Elkabes S (2005). Plasma membrane calcium ATPase deficiency causes neuronal pathology in the spinal cord: a potential mechanism for neurodegeneration in multiple sclerosis and spinal cord injury. FASEB J..

[43] Hu JZ (2015). The effect of estrogen-related receptor alpha on the regulation of angiogenesis after spinal cord injury. Neuroscience.

[44] Zhang M (2015). Bone marrow mesenchymal stem cell transplantation retards the natural senescence of rat hearts. Stem Cells Transl. Med..

[45] G. Zheng Y (2018). Monascin inhibits IL-1β induced catabolism in mouse chondrocytes and ameliorates murine osteoarthritis. Food Funct..

[46] Li P, Kaslan M, Lee SH, Yao J, Gao Z (2017). Progress in exosome isolation techniques. Theranostics.

[47] Wu, J. C., Luo, S. Z., Liu, T., Lu, L. G. and M. Y. Xu, linc-SCRG1 accelerates liver fibrosis by decreasing RNA-binding protein tristetraprolin. *FASEB J*. 10.1096/fj.201800098RR (2018). 10.1096/fj.201800098RR 30226813

