# Supplementary figures and images for: Hypoxia preconditioning promotes bone marrow mesenchymal stem cells survival by inducing HIF-1α in injured neuronal cells derived exosomes culture system
Source: Cell Death Dis. 2019 Feb 12;10(2):134. doi: 10.1038/s41419-019-1410-y (PMC6372680; doi:10.1038/s41419-019-1410-y)

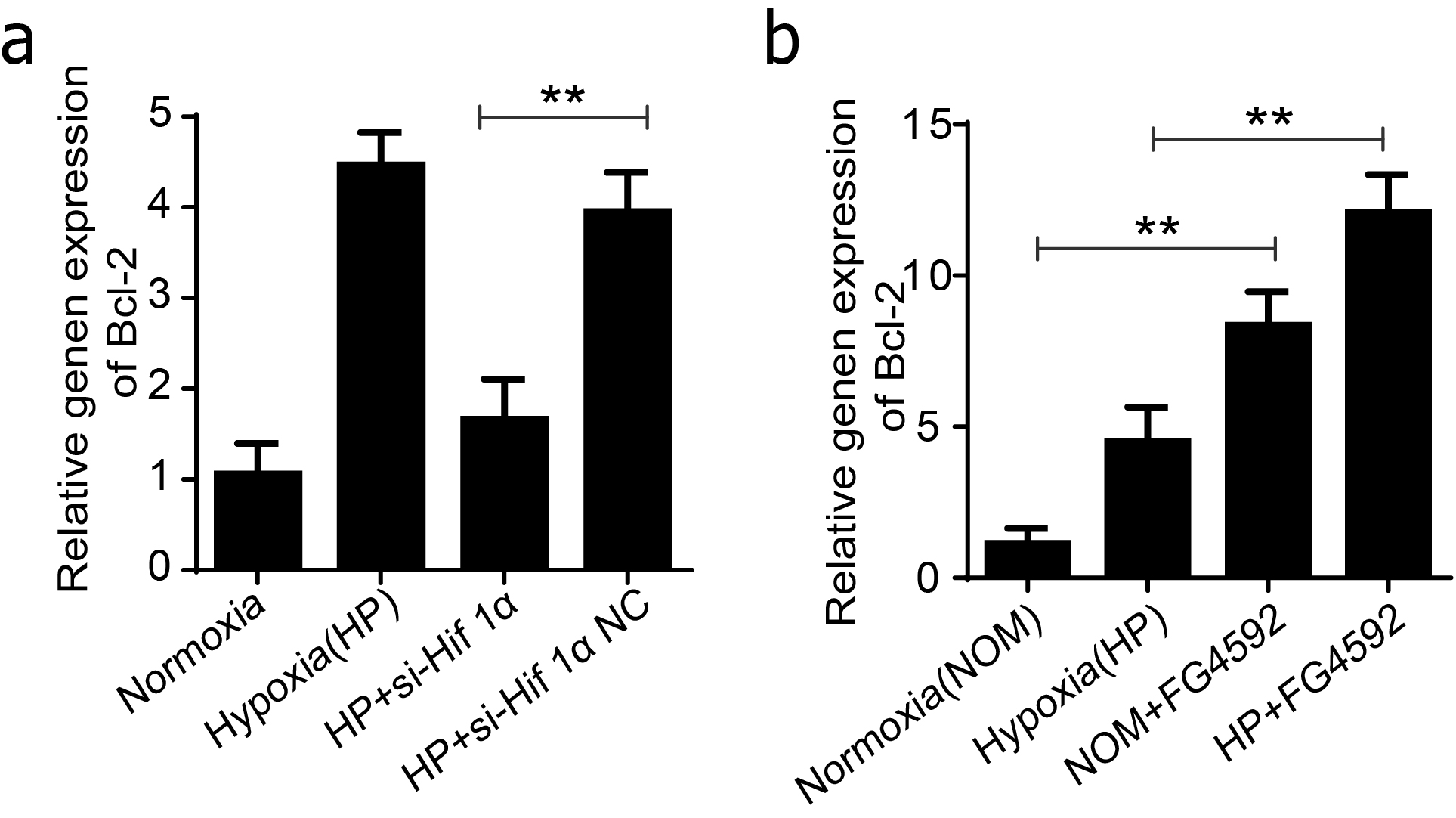

Supplement: Supplementary file 1 — supplemental data [file 41419_2019_1410_MOESM1_ESM.jpg]
