# Supplementary material for: Hypoxia preconditioning promotes bone marrow mesenchymal stem cells survival by inducing HIF-1α in injured neuronal cells derived exosomes culture system
Source: Cell Death Dis. 2019 Feb 12;10(2):134. doi: 10.1038/s41419-019-1410-y (PMC6372680; doi:10.1038/s41419-019-1410-y)
Supplement: Supplementary file 2 — Supplemental figure legends [file 41419_2019_1410_MOESM2_ESM.docx]

Figure legends

Supplemental data1: (a, b) the transcripts of HIF-1 target genes of Bcl-2 were measured by RT-PCR, the data showed that the transcription level of the Bcl-2 gene increases with the increase of HIF-1α.
